# Circulating amino acids and Type 2 diabetes in a Latin American population-based cohort

**DOI:** 10.1186/s12933-026-03146-8

**Published:** 2026-03-26

**Authors:** Laura Huidobro, Sebastián Cofre, José L. Santos, Franco Godoy, Fabio Paredes, Claudia Bambs, Sandra Cortés, John Connolly, Hui-Qi Qu, Hakon Hakonarson, Catterina Ferreccio

**Affiliations:** 1 Department of Preclinical Sciences, Faculty of Medicine, Universidad Católica del Maule, Talca, Chile; 2 Faculty of Medicine, School of Public Health, Pontificia Universidad Católica de Chile, Santiago, Chile; 3 Advanced Center for Chronic Diseases (ACCDiS) Research Center in Priority Areas, Pontificia Universidad Católica de Chile, Santiago, Chile; 4 Center for Cancer Prevention (CECAN), Research Center in Priority Areas, Pontificia Universidad Católica de Chile, Santiago, Chile; 5 Faculty of Health Sciences, School of Nutrition and Dietetics, Universidad Católica del Maule, Talca, Chile; 6 Institute for Sustainability and IdiSNA, Navarra Institute for Health Research (IdiSNA), Pamplona, Spain; 7 Department of Health Sciences, Navarra Institute for Health Research (IdiSNA), Pamplona, Spain; 8 Department of Nutrition, Diabetes and Metabolism, School of Medicine, Pontificia Universidad Católica de Chile, Santiago, Chile; 9 Faculty of Mathematics, Pontificia Universidad Católica de Chile, Santiago, Chile; 10 Center for Sustainable Urban Development (CEDEUS), Santiago, Chile; 11 The Center for Applied Genomics, Children’s Hospital of Philadelphia, Philadelphia, PA 19104 USA; 12 Division of Human Genetics, Children’s Hospital of Philadelphia, Philadelphia, PA 19104 USA; 13 Department of Pediatrics, The Perelman School of Medicine, University of Pennsylvania, Philadelphia, PA 19104 USA; 14 Division of Pulmonary Medicine, Children’s Hospital of Philadelphia, Philadelphia, PA 19104 USA; 15 Faculty of Medicine, University of Iceland, 101 Reykjavik, Iceland; 16 Chile’s Institute of Public Health, Ministry of Health, Santiago, Chile

**Keywords:** Type 2 diabetes, Metabolomics, Branched-chain amino acids

## Abstract

**Background and aims:**

Metabolomics enables the identification of circulating biomarkers for Type 2 diabetes (T2D). Most studies of circulating amino acids and T2D are from European and Asian, few from Latin America. We aimed to evaluate plasma amino acids and risk of T2D in an agricultural population from Molina County, Central Chile.

**Methods:**

MAUCO is a population-based prospective cohort of 9462 participants aged 38 to 74 years at enrollment in 2014. From 2000 participants (47% women) selected for metabolomic analysis 1738 had enough serum sample for this study. We quantified circulating branched-chain amino acids (BCAAs), as well as phenylalanine, tyrosine, alanine, glutamine, glycine, and histidine using nuclear magnetic resonance, while T2D was diagnosed according to the American Diabetes Association guidelines at enrollment and follow-up. We analyzed the association of the circulating amino acids on T2D prevalence and incidence, using multiple logistic and Cox regression models adjusted by sex, age, education, body mass index, smoking, alcohol consumption, physical activity, and Mediterranean diet score.

**Results:**

In the cross-sectional analysis, plasma BCAAs (aOR 2.1; 95% CI 1.80–2.50), and plasma alanine (aOR 1.57; 95% CI 1.37–1.80) were associated with T2D, while histidine, glycine, and glutamine were inversely associated with T2D risk (aOR 0.80; 95% CI 0.69–0.91; 0.68; 95% CI 0.57–0.80, and 0.64; 95% CI 0.56–0.74, respectively). After a median follow-up of 4.3 years, we diagnosed 127 (10.5% incidence) new T2D cases. Prevalence and incidence analysis yielded a similar pattern, but only high isoleucine reached statistical significance in the incidence of T2D (aHR 1.31; 95% CI 1.10–1.56).

**Conclusion:**

Elevated plasma BCAA concentrations in Chilean adults were significantly associated with the prevalence of T2D, while only high isoleucine was associated with the incidence of T2D. These findings could inform risk stratification by specific metabolic mechanisms and guide future research on targeted interventions.

**Supplementary Information:**

The online version contains supplementary material available at 10.1186/s12933-026-03146-8.

## Research insights

**What is currently known about this topic?**

- Metabolomics can help understanding the physiopathology of T2D and contribute to preventative and therapeutics measures.
- Elevated plasma concentrations of branched-chain, aromatic amino acids and low levels of glycine have been consistently associated with insulin resistance and an increased risk of T2D in European and Asian populations.

**What is the key research question?**

- Are circulating levels of branched-chain and branched-chain related amino acids associated with the prevalence and incidence of T2D in a Latin American population from the MAUCO cohort, Chile?.

**What is new?**

- There is a significant cross-sectional association between higher BCAA concentrations and alanine, and T2D. We found an inverse association and potential protective role, of glycine, glutamine, and histidine, on T2D risk.
- Plasma levels of isoleucine were associated with higher incidence of T2D strengthening its potential risk role.

**How might this study influence clinical practice?**

- Based on these findings research directions on amino acid profiles associated with T2D risk should focus on risk stratification by specific metabolic mechanisms and thus provide stronger insights for personalized interventions with diet or precision drugs.

## Background

Metabolomic techniques applied to biofluids such as plasma offer the possibility to interrogate a wide range of metabolites involved in chronic diseases development. The study of specific metabolites may provide valuable information on prediction, diagnosis, and disease progression [1]. Among multiple small-size compounds available in targeted metabolomic panels, free plasma amino acids constitute a group of relevant intermediary metabolites that have been used as biomarkers of both monogenic diseases (e.g., maple syrup urine disease, phenylketonuria, among others), as well as chronic diseases such as T2D [2]. In the last years, the branched-chained amino acids (BCAA) such as valine, leucine and isoleucine have focused attention as circulating biomarkers for T2D prediction [3]. BCAAs are amphipathic essential amino acids that have been widely studied in relation to muscle metabolism and protein synthesis [3]. After a rich-protein meal, absorbed amino acids are present in the portal vein in proportion to the protein composition of the meal. However, BCAA are poorly retained in the liver due to the low hepatic expression of the branched-chain-amino-acid aminotransferase (BCAT), the first enzyme involved in BCAA catabolism. Then, a high proportion of free BCAA are normally present in the plasma leaving the liver, being such BCAA preferentially metabolized in the skeletal and cardiac muscle tissue [4]. In the muscle, BCAAs enter amino transference reactions and specific degradation pathways that regulate the release of alanine and glutamine (main amino acids leaving the muscle into circulation), as well as a significant amount of BCAA-derived keto acids [3, 4]

As early as in 1969, it was reported that plasma levels of aromatic amino acids phenylalanine and tyrosine, and BCAAs, were higher in patients with obesity compared to matched normal-weight individuals. In contrast, plasma glycine levels showed the opposite pattern [5]. After this initial study, the use of metabolomic techniques repeatedly confirmed such a positive association between BCAA and aromatic amino acids with higher risk of obesity, insulin resistance and T2D, and an inverse association with plasma glycine levels [2], suggesting that they influence insulin sensitivity/secretion and have a causative role in T2D [6].

Most of the studies in the field of metabolomics linked to T2D have been conducted in European and Asian populations [7]. Therefore, prospective studies are needed in the Latin American population to identify potential biomarkers of chronic diseases using plasma metabolomic profile. The ELSA cohort in Brazil showed that elevated levels of BCAAs were associated with a T2D hazard ratio (HR) of 2.24 (95% CI 1.24–4.03) in men, and a T2D HR of 1.94 (95% CI 1.07–3.50) in women [8]. Likewise, Rivas- Tumanyan et al. [9] in another cohort of Puerto Rican adults, observed that higher baseline BCAAs level were associated with a higher prevalence of T2D (OR 1.46; 95% CI: 1.34–1.59) and a higher risk of T2D incidence (IRR 1.24; 95% CI: 1.13–1.37).

The Maule Cohort (MAUCO) is the first prospective, agricultural population-based cohort study established to analyze the natural history of cardiovascular disease (CVD) and cancer in central Chile [10]. The agricultural environment can significantly influence metabolic risk through specific environmental and occupational exposures, most notably chronic contact with agrochemicals. These factors, combined with a 92.7% rate of low physical activity, create a specific metabolomic profile that is essential for understanding the etiology of metabolic dysfunction and progression of T2D [36]. Furthermore, the high geographic stability and low migration rates of this district ensure high-fidelity longitudinal follow-up, which is essential for tracking the natural history of chronic diseases. Likewise, the combination of sociodemographic vulnerability, characterized by lower schooling levels and high public health insurance reliance, offers a critical perspective on health disparities in Latin America [36]. Consequently, this population is uniquely positioned to bridge gaps in epidemiological research regarding the intersection of environmental factors, metabolome and metabolic health.

However, these studies are focused on BCAAs and do not consider other relevant amino acids that may influence BCAAs metabolism. The purpose of this study was to evaluate the associations between T2D and plasma concentrations of BCAA with a panel of relevant amino acids in a population-based cohort developed in an agricultural county in central Chile [10], thus identifying biomarkers that predict T2D in a Latino population living in South America.

## Methods

### Aim of the study

We aimed to evaluate plasma amino acids and risk of T2D in an agricultural population from Molina County, Central Chile.

### Study design

Our study was conducted in the Maule Cohort (MAUCO) in the agricultural county of Molina, Maule Region in Chile. The MAUCO study protocol has been published elsewhere [10] and defines the following selection criteria: to be a resident of Molina for at least 6 months and without plans to move for the next 3 years, aged 38 to 74 years, and being able to consent autonomously [10]. Exclusion criteria were having a terminal disease with a projected life expectancy of under 12 months or being unable to reach the outpatient clinic where the study was being held.

From the 9,462 participants enrolled between December 2014 and June 2020 in the cohort, we intended a sample of 2,000 from whom 1,738 were suitable for analysis (47% women; aged 38–74 years).

All participants had already signed informed consent, where they accepted the use of their samples for omic assays as well as access to their clinical records, and the project was approved by the Scientific Ethics Committee of Pontificia Universidad Católica de Chile, and the Ethics Committee of the Servicio de Salud del Maule.

### Type 2 diabetes assessment

T2D was defined according to American Diabetes Association (ADA) [11] guidelines, as an elevated glucose level at any time during the study (8 h fasting glucose ≥ 126 mg/dL or a random plasma glucose ≥ 200 mg/dL with classic symptoms of hyperglycemia), or self-reported diagnosis of diabetes, or use of hypoglycemic drugs, or as registered in the clinical record by medical staff at recruitment and visits during 5-year follow up. To estimate diabetes incidence and Cox analysis, 309 cases of diabetes included at recruitment were excluded, and only cases diagnosed in one of the follow-up visits were considered. Similarly, 235 participants were excluded because they had only one baseline measurement and no follow-up.

### Plasma amino acid profiling

Fasting blood samples were collected by trained personnel. Amino acid quantification was performed in plasma samples obtained from whole-blood EDTA-containing tubes and stored at -80 °C until laboratory analysis using nuclear magnetic resonance (NMR) spectroscopy via the high-throughput NMR metabolomics platform (Nightingale Health Ltd., Helsinki, Finland) [12–14]. After reviewing the technical details of the metabolic biomarker data [15], and conducted an exhaustive quality-control analysis, the NMR platform estimated absolute concentrations of 168 metabolites, covering free amino acid measurement of BCAAs (valine, leucine, isoleucine), as well as phenylalanine, tyrosine, alanine, glutamine, glycine and histidine. The complete analysis of metabolites also included glycolysis/gluconeogenesis metabolites, ketone bodies, non-esterified fatty acids (NEFAs), inflammatory biomarkers, and apolipoprotein/lipoprotein-related measurements, not considered in the present study. No data loss was observed during NMR analysis among the metabolites analyzed.

### Cardiovascular risk factors and anthropometry

Levels of triglycerides, total cholesterol, LDL-cholesterol, HDL-cholesterol and VLDL-cholesterol were determined by enzymatic colorimetric assay at the Molina Hospital located in the Maule district [10]. The quantification of plasma lipid profiles allowed us to calculate the Framingham Cardiovascular Risk Score. Body weight, height, and waist circumference were measured by trained personnel, and Body Mass Index (BMI) was calculated.

### Lifestyle variables

Daily alcohol consumption (g/day), smoking status, and physical activity were assessed using standardized instruments. We employed the brief version of the Alcohol Use Disorders Identification Test (AUDIT), adapted for the Chilean population, to assess alcohol consumption [16]. Levels of moderate, vigorous, and transport-related physical activity were quantified using the validated questionnaire called Global Physical Activity Questionnaire version 2 (GPAQ v2) [17]. Following standard protocols, the sample was dichotomized into inactive (< 600 MET/min/week) and active (≥ 600 MET/min/week) individuals.

Adherence to the Mediterranean diet score (MDS) was evaluated through a food frequency questionnaire (FFQ) comprised of 28 items [18]. The MDS was subsequently calculated, ranging from 0 to 14, with lower scores indicating low adherence and higher scores indicating greater adherence to the Mediterranean dietary pattern. The specific components of the MDS are detailed in the Table S1.

### Statistical analysis

A descriptive analysis of the baseline characteristics of the participants was carried out providing summary statistics (Table 1). Continuous data were compared using Student’s t-tests for normally distributed variables and Wilcoxon rank-sum tests for non-parametric distributions. For categorical data, chi-squared tests were employed to examine associations. For each amino acid considered in this study, values were standardized within the analytic sample by subtracting the mean and dividing by the standard deviation (z-score). Regression coefficients and effect sizes are therefore interpretable per 1-SD (standard deviation) increase in the metabolite level. We used multiple logistic regression to assess the association of amino acids with prevalent diabetes at baseline, and a Cox proportional hazards model for the incidence of diabetes during the follow-up period. Besides analyzing standardized amino acid levels, we also analyzed them in quartiles to explore non-linear and lineal trend associations. For confounding control and covariate selection, we selected covariates a priori using subject-matter knowledge and prior evidence on common causes of plasma amino acids and T2D. Specifically, we adjusted for: age and sex (demographic confounding), adiposity (BMI), education level (as socioeconomic position), and lifestyle/dietary factors (smoking status, alcohol intake, physical activity, and MDS). Covariates were not chosen via stepwise procedures or p-value screening. We avoided adjusting for downstream variables on the causal pathway (e.g., glycemic biomarkers) to minimize overadjustment. For both logistic and Cox regressions, we fitted three different models to adjust for confounders: Model 1 was adjusted by age and sex; Model 2 added education and BMI on top of model 1; and Model 3 included all the above plus MDS, alcohol consumption, smoking status and physical activity. We corrected for multiple testing across 10 amino-acid exposures using a Bonferroni procedure. Statistical significance was set at a two-tailed *p* value < 0.5. We also generated log rank curves to illustrate T2D incidence for each and all BCAA levels. We performed all analyses using the Rstudio (R) software version 4.4.1.

**Table 1:** Health characteristics and amino acids levels at study enrollment in 1738 MAUCO participants

	Women n = 826	Men n = 912	*p*- value for sex differences	Bonferroni Corrected *p*-value
Age at recruitment (y)	56.0 (49.0, 64.0)	54.0 (47.0, 61.0)	< 0.001	0.026
Education years, n (%)			0.598	1
≤ 8 y	378 (45.8)	417 (45.7)		
9–12 y	322 (39.0)	371 (40.7)		
≥ 13 y	126 (15.2)	124 (13.6)		
Smoking status, n (%)			< 0.001	0.026
Never smoker	461 (56.7)	334 (36.7)		
Past smoker	143 (17.6)	245 (27.4)		
Current smoker	212 (25.7)	321 (35.9)		
Daily alcohol intake (g/day)	1.3 (0.6, 2.6)	3.8 (1.3, 10.2)	< 0.001	0.026
Type 2 diabetes, n (%)	168 (20.3)	134 (14.7)	0.003	0.078
Non-alcoholic fatty liver disease n (%)	426 (46.7)	478 (57.8)	0.299	1
BMI (kg/m^2^)	28.6 (25.3, 32.2)	28.4 (25.8, 31.3)	0.431	1
Glycemia (mg/dL)	93.8 (87.0, 103)	95.3 (89.0, 102)	0.013	0.338
Abdominal obesity, n (%)	755 (91.3)	672 (73.7)	< 0.001	0.026
Low HDL- cholesterol, n(%)	426 (46.7)	478 (57.8)	< 0.001	0.026
Total cholesterol (mg/dL)	192.0 (168.7, 222.0)	188.2 (163.6, 214.4)	0.009	0.234
HDL-cholesterol (mg/dL)	48 (40, 55)	41 (34, 48)	< 0.001	0.026
LDL-cholesterol (mg/dL)	113.7 (94.6, 139.7)	112.9 (91.5, 136.6)	0.160	1
Triglycerides (mg/dL)	126.0 (90.6, 180.4)	145.1 (97.6, 209.8)	< 0.001	0.026
Framingham Risk Score	2.0 (1.0, 5.0)	10.0 (6.0, 16.0)	< 0.001	0.026
Mediterranean Diet Score (MDS)	5.5 (4.5, 6.5)	4.5 (4.0, 6.0)	< 0.001	0.026
Alanine (μmol/L)	0.35 (0.30, 0.41)	0.38 (0.33, 0.44)	< 0.001	0.026
Glycine (μmol/L)	0.60 (0.53, 0.66)	0.62 (0.55, 0.68)	< 0.001	0.026
Histidine (μmol/L)	0.08 (0.07, 0.08)	0.08 (0.08, 0.09)	< 0.001	0.026
Total BCAA (μmol/L)	0.36 (0.32, 0.41)	0.42 (0.38, 0.47)	< 0.001	0.026
Isoleucine (μmol/L)	0.05 (0.04, 0.06)	0.06 (0.05, 0.07)	< 0.001	0.026
Leucine (μmol/L)	0.10 (0.09, 0.11)	0.12 (0.11, 0.14)	< 0.001	0.026
Valine (μmol/L)	0.21 (0.19, 0.24)	0.23 (0.21, 0.26)	< 0.001	0.026
Phenylalanine (μmol/L)	0.05 (0.05, 0.06)	0.06 (0.05, 0.06)	< 0.001	0.026
Glutamine (μmol/L)	0.23 (0.18, 0.29)	0.19 (0.16, 0.24)	< 0.001	0.026
Tyrosine (μmol/L)	0.06 (0.06, 0.07)	0.07 (0.06, 0.08)	< 0.001	0.026

Values are presented as n (%) for categorical variables and median (interquartile range) for continuous variables. Group comparisons were performed using the Chi-square test for categorical variables and the Wilcoxon rank-sum test for continuous variables

The were not losses among the metabolites analyzed.

## Results

### Characteristics of the participants

We analyzed 826 (47.5%) women and 912 (52.5%) men participants, median age of 55 years, 45.7% had less than 8 school years (Table 1). Statistically significant differences were reported for most cardiovascular parameters between women and men. We observed higher levels of alcohol intake, smoking, triglycerides, Framingham score, blood pressure, blood glucose, HDL Cholesterol and MDS in men. Conversely, women had significantly higher age, higher levels of total cholesterol and presence of central obesity. There were no significant differences between sexes in terms of education, BMI, LDL cholesterol, and steatotic liver disease (SLD). The prevalence of T2D was higher in women. Differences were also observed in plasma concentrations of all amino acids analyzed when grouped by sex, with higher levels in all amino acids in men, except for glutamine which was higher in women (Table 1). After Bonferroni adjustment, prevalence of T2D and blood levels of glycemia and total cholesterol were no longer different between sexes.

### BCAAs, amino acids and their associations with prevalent and incident cases of T2D cross-sectional analysis

**Fig. 1:**
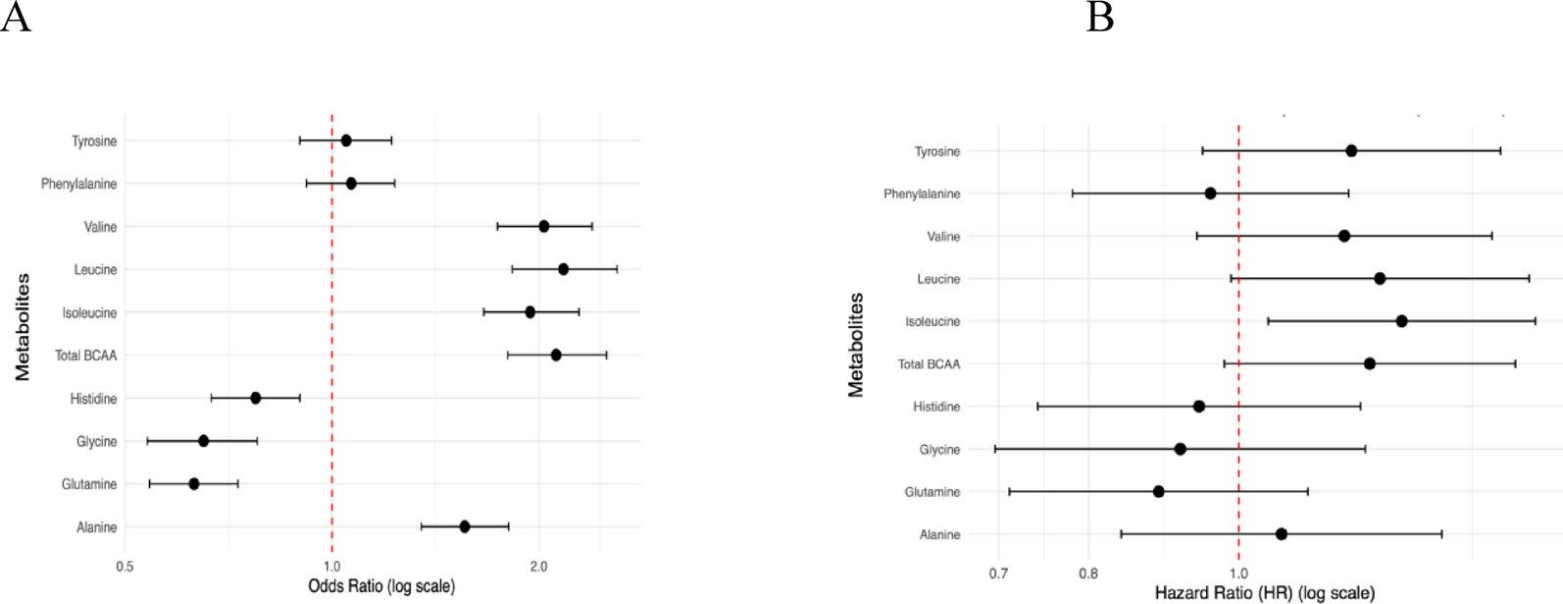
Association between AA at recruitment to MAUCO cohort and prevalent **A** and incident **B** T2D in 1738 MAUCO participants

Adjusted odds ratio (aOR) for T2D per 1-SD increase in amino acid concentrations was 1.95 (95% CI 1.68–2.28) for isoleucine, 2.06 (95% CI 1.77;2.39) for valine, 2.17 (95% CI 1.85- 2.57) for leucine, 2.14 (95% CI 1.83; 2.51) for total BCAA, and 1.57 (95% CI 1.37–1.80) for alanine (Fig. 1A). The results also highlight significant inverse associations between 1-SD change in plasma levels of histidine (aOR 0.80, 95% CI 0.69–0.91), glutamine (aOR 0.64, 95% CI 0.56–0.74) and glycine (aOR 0.68, 95% CI 0.57–0.80) with T2D. The associations with isoleucine, valine, BCAA, tyrosine and phenylananine remained significant after Bonferroni post hoc test to account for multiple comparisons (Table S2). Stratified analysis showed no interaction for BCAA and sex, liver status, central obesity and obesity (Tables S3 to S6).

### Incident analysis

**Fig. 2:**
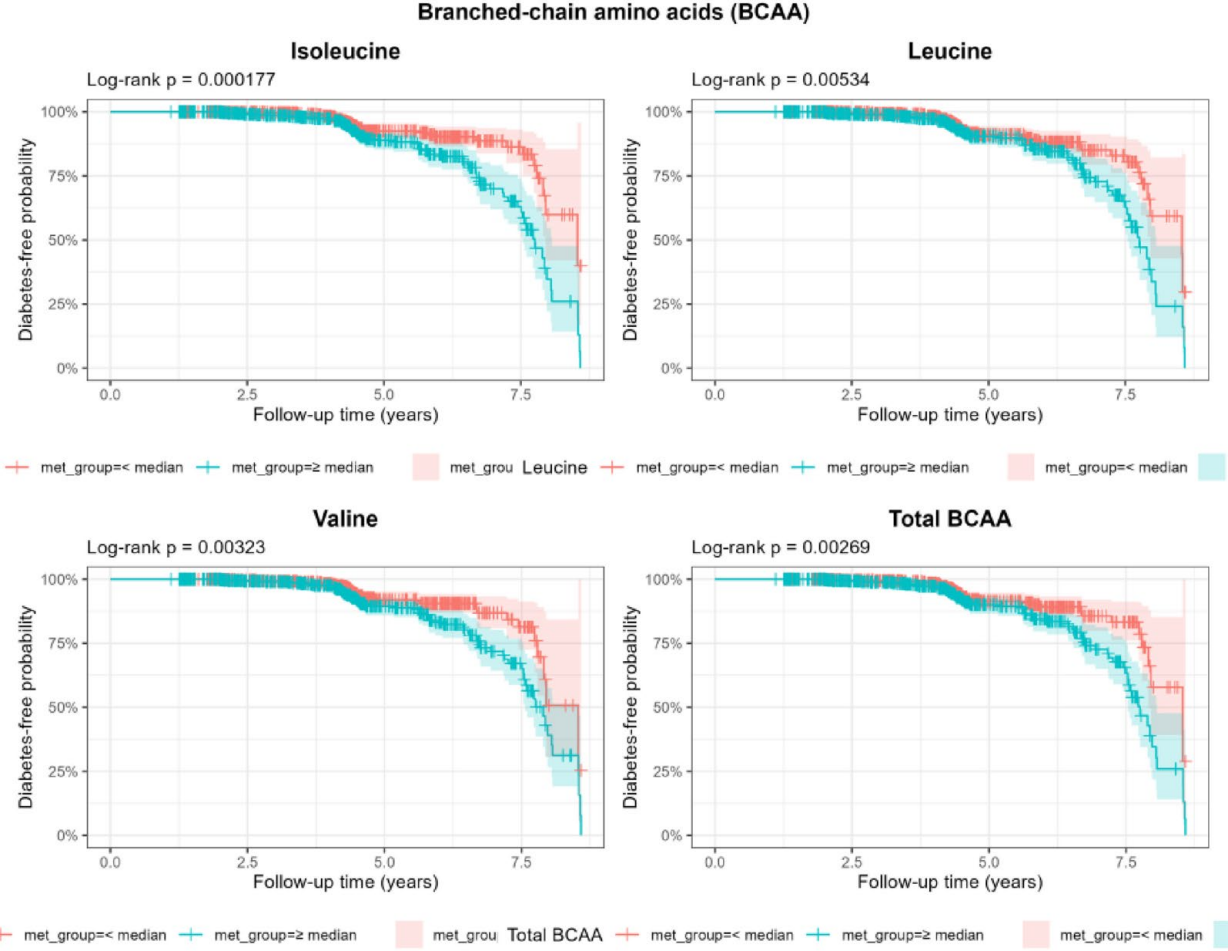
T2D incidence according to median concentrations of BCAA in 1194 participants of MAUCO cohort

After a median follow-up of 4.3 years, we diagnosed 127 new cases of T2D. Associations between AA and T2D are shown in Fig. 1B. The log-rank curves for T2D survival were significant for all three BCAA (Fig. 2). We observed an increased risk (aHR) of diabetes of 1.31 (95% CI 1.10–1.56) per 1-SD increase in isoleucine, 1.27 (95% CI 1.03–1.55) for leucine, 1.24 (95% CI 1.03–1.52) for BCAA, and 1.23 (95% CI 1–1.50) for tyrosine. After Bonferroni correction, only isoleucine remained statistically significant (Table S7).

(A) aOR per 1-SD change in plasma concentrations of amino acids. (B) aHR per 1-SD change in plasma concentrations of amino acids. Both models were adjusted by sex, age, education, BMI, alcohol consumption, physical activity, smoking status and MDS.

Model was adjusted by sex, age, education, BMI, alcohol consumption, physical activity, smoking status and MDS.

## Discussion

After ELSA and Puerto Rican cohorts, this is the third cohort study to analyze the association between plasma branched-chain amino acid (BCAA) and BCAA-related amino acids concentrations and the risk of developing T2D in a Latin American population.

We diagnosed 127 new cases of T2D 10.5% of incidence) after a mean of 4.3-year follow-up (IQR 3,1–5.0). These findings are comparable to those reported by de Almeida-Pititto et al. [8], who observed a 7.8% incidence of T2D after a median follow-up period of 3.9 years in a Brazilian population. It also aligns with those previously reported in a systematic review led by Ilic et al. [27], who found that the incidence was 6.2% in the Andean Latin American population in 2019, slightly higher in the female population.

This study shows a strong cross-sectional association between circulating BCAA levels and T2D in Chilean adults, with a positive association for alanine and inverse associations for glycine, histidine, and glutamine. When these associations were tested for the incidence rate of T2D after a 4.3-year follow-up, only plasma leucine remained statistically significant. These results are broadly consistent with previous international studies [7]. Our report is one of the few epidemiological studies conducted in Latin America to assess the association between plasma BCAA levels and T2D, along with studies in Puerto Rico and Brazil [8, 9]. Regarding cross-sectional BCAA-T2D associations, our study shows odds ratios very similar to those reported in the Puerto Rico study [9]. For incident T2D, our results also show a similar general pattern in terms of the magnitude of association between plasma BCAA and new cases of T2D compared to a cohort in Brazil [8] and Puerto Rico [9], although showing small differences possibly derived from disparities population backgrounds, as well as in the selection of covariates in statistical models and time of follow-up.

### Amino-acid predictors of incident T2D and implications for disease progression

Our full adjusted model only found a relationship between isoleucine plasma levels and the incidence of T2D. Likewise, Rivas-Tumayan et al. [9] reported a positive association between an increase in plasma isoleucine concentrations by 1-SD and the incidence of T2D in a Puerto Rican population. Another study, led by Flores-Guerrero et al. [19], also found associations between isoleucine levels and a higher incidence of diabetes in the Dutch population. In a systematic review with meta-analysis (SR-MA) Morze et al. [7] reported that 1-SD increase in plasma isoleucine was associated with 54% higher risk of T2D (RR 1.54, 95% CI 1.36–1.74) with data of nineteen cohorts. The accumulated evidence underscores the importance of metabolic profiles, especially amino acids, in understanding the pathogenesis and assessing the risk of T2D.

Another noteworthy finding is that men exhibited higher plasma concentrations of amino acids—except for glycine—compared with women. One plausible explanation center on men’s body composition, as they typically have greater muscle mass than women, as well as differences in dietary intake of protein-rich foods [20].

Although the precise pathophysiology of the relationship between plasma BCAA and T2D is still under investigation, it is suggested that BCAA could impair insulin signaling through mTOR kinase activation and the accumulation of toxic intermediates, leading to pancreatic beta-cell dysfunction and exhaustion [20, 21]. Moreover, some studies highlight that alterations in amino acid metabolism may precede hyperglycemia, being BCAA levels predictors of future glycemia in the general population [5, 22–24]. On the other side, it has been hypothesized that pre-existing undetected changes in insulin resistance may have an impact on BCAA levels, potentially through the process of hyperglycemia-induced suppression of adipose tissue expression of genes involved in branched-chain amino acid oxidation [25–27].

Mechanistically, elevated circulating BCAAs may reflect impaired peripheral BCAA catabolism, leading to mitochondrial overload, accumulation of branched-chain ketoacids, and impaired insulin signaling [2]. The inverse associations for glycine and histidine are also biologically plausible: glycine depletion has been linked to increased oxidative stress and diversion toward glutathione synthesis [28], while lower histidine may limit carnosine formation and reduce buffering and anti-glycation capacity [29]. Together, these patterns are consistent with impaired oxidative metabolism and early insulin resistance, supporting a role for BCAA-related metabolic pathways in the development of T2D.

Several contextual factors may help explain the pattern of associations observed. Despite men having higher amino-acid concentrations, their lower prevalence of T2D compared with women likely reflects sex differences in adiposity distribution, muscle mass, and lifestyle exposures [30]. The strong cross-sectional associations for multiple amino acids, contrasted with the longitudinal finding that only isoleucine remained significant after correction, suggest that most amino-acid alterations reflect existing metabolic dysregulation rather than early predictors of disease [23]. Isoleucine may be the most informative BCAA in this cohort, consistent with prior studies showing that it often displays the strongest association with incident T2D [17]. The inverse association of glutamine, which was higher in women, may reflect sex-specific amino-acid turnover or differences in dietary intake [30]. The low Mediterranean Diet Score observed in this cohort, combined with the high prevalence of abdominal obesity and steatotic liver disease, may create a metabolic environment that amplifies disturbances in amino-acid metabolism [31]. Overall, these findings suggest that while broad amino-acid alterations characterize existing T2D, isoleucine may be the most informative marker of earlier metabolic change.

From a population perspective, these findings are particularly relevant in the Chilean and broader Latin American context. MAUCO participants live in an agricultural county with dietary patterns that often combine high intake of refined carbohydrates, red and processed meats, and relatively low adherence to a traditional Mediterranean pattern, which may increase dietary BCAA load and exacerbate underlying insulin resistance [32]. Chile has completed a process of epidemiologic transition, with a rising influence of unhealthy dietary habits impacting a growing prevalence of obesity and comorbidities. These elements, together with a particular population structure and genetic admixture, may influence the contribution of metabolomic biomarkers in the development of T2D compared to other populations. Then, it is important to conduct epidemiologic studies across diverse countries, including Chile and Latin American countries, to refine metabolomic signatures of chronic diseases.

In addition, Chilean populations have a substantial Amerindian ancestry component, which has been associated with higher susceptibility to central obesity, NAFLD and type 2 diabetes, and may influence amino-acid handling through differences in adiposity distribution and mitochondrial function [33]. Although we did not directly assess genetic ancestry or tissue-specific BCAA catabolic capacity (e.g., BCAT/BCKDH activity), the very high prevalence of abdominal obesity and steatotic liver disease in this cohort suggests a metabolic milieu in which impaired BCAA oxidation and altered amino-acid flux could be particularly pronounced [34]. Thus, our results may reflect the interaction between a characteristic Latin American cardiometabolic risk profile and BCAA-related pathways, underscoring the importance of studying these mechanisms in diverse, non-European populations.

### Methodological considerations of NMR-based amino-acid profiling

Regarding metabolomics analysis techniques, most of the studies analyzed in this discussion used NMR-based techniques. NMR is inherently quantitative and highly reproducible and often used in high-throughput metabolomics, allows for the simultaneous identification and quantification of amino acids and other metabolites that are part of central metabolism, such as organic acids and sugars [15, 35]. NMR is expensive and available only for investigative purposes; therefore, in the meanwhile, our efforts must focus in finding out critical cut-off levels for early diagnosis and more precise metabolomic signatures to detect higher risk population before they develop hyperinsulinemia or hyperglycemia.

The present study has several strengths. First, our study evaluates a more comprehensive panel of relevant amino acids including BCAAs and AAs, being these metabolites key to understanding the pathophysiology of T2D. Second, by using metabolomic techniques on biofluids such as plasma, the study has the potential to identify early biomarkers for the prediction, diagnosis, and progression of T2D. The discovery of these markers is a valuable objective for identifying individuals at higher risk, which could lead to more innovative and targeted prevention strategies. And third, our study was conducted in a Latino population living in South America, enabling us to gain insight into how this epidemiological association manifests in populations other than those of European or Asian origin, which have been the subject of extensive biomedical research. Nevertheless, a significant weakness of this study is the reliance on a single set of plasma BCAA and AA measurements, taken only at baseline. Additionally, the scope of our study did not encompass the measurement of metabolites associated with environmental exposures, such as pesticides or organophosphates. Thus, there is a possibility for residual confusion that must be considered during the interpretation of the findings. On the other hand, we couldn't track changes in the metabolomic profile across the follow-up and its interaction with other metabolites.

We should also acknowledge that our study is only applicable to a semi-urban population and that residual confounding may be present, particularly because metabolic control of diabetes among individuals with diabetes could not be considered when assessing T2D prevalence. In addition, although constraints regarding variable measurements are possible in our study, our team is highly trained in data collection and management; therefore, we do not expect these limitations to have a substantial impact on the results.

Future research in this cohort will incorporate new events and will integrate additional metabolites to calculate complex predictive risk scores based on the stratification of the population, as well as identification of interactions between specific metabolites, diet, and disease.

## Conclusions

There is consistent cross-sectional association between BCAA and Alanine, and T2D, as well as a negative association between Glycine, Glutamine and Histidine, suggesting a possible protective role. Likewise, Isoleucine showed an independent positive association with T2D incidence. Our epidemiologic research shows that elevated plasma BCAA concentrations were strongly associated with T2D in Chilean adults. These findings could guide future research toward targeted intervention strategies relevant to precision medicine for T2D.

## Supplementary Information

Below is the link to the electronic supplementary material.

Supplementary Material 1

Supplementary Material 2

Supplementary Material 3

## Data Availability

Data are available upon reasonable request, following approval of the analysis plan from the study researchers. For more information on requesting data, please to visit: https://www.mauco.org/

## Abbreviations

aHR: Adjusted hazard ratio
aOR: Adjusted odds ratio
BCAA: Branched chain amino acids
BCAT: Branched chain amino acid aminotransferase
MDS FFQ: Food frequency questionnaire Mediterranean diet score
NMR: Nuclear magnetic resonance
SLD: Steatotic liver disease
T2D: Type 2 diabetes mellitus
