# Supplementary material for: Circulating amino acids and Type 2 diabetes in a Latin American population-based cohort
Source: Cardiovasc Diabetol. 2026 Mar 26;25:116. doi: 10.1186/s12933-026-03146-8 (PMC13054996; doi:10.1186/s12933-026-03146-8)
Supplement: Supplementary file 1 — Supplementary Material 1 [file 12933_2026_3146_MOESM1_ESM.docx]

**Supplemental Material**

Table S1: Items for scoring of Mediterranean Diet Score (MDS)

|  | Score item | Score units | 1.0 points | 0.5 points | 0 points |
| --- | --- | --- | --- | --- | --- |
| 1 | Vegetables (excluding potatoes) | Servings per day | ≥3 | 1-3 | <1 |
| 2 | Legumes and nuts | Servings per week | ≥2 | 1-2 | <1 |
| 3 | Fruits | Servings per day | >2 | 1-2 | <1 |
| 4 | Whole grains | Servings per day | ≥2 | 1-2 | <1 |
| 5 | Lean meat | Servings per week | >4 | 2-4 | <2 |
| 6 | Fish and shellfish | Servings per week | >2 | 1-2 | <1 |
| 7 | Fatty meat and processed meat | Servings per week | <1 | 1-2 | >2 |
| 8 | Full fat dairy products not fermented | Servings per day | <1 | 1-2 | ≥2 |
| 9 | Low-fat and fermented dairy products | Servings per day | ≥2 | 1-2 | <1 |
| 10 | Vegetable oils | Teaspoons per day | 4–8 | 2-4 | >8 or <2 |
| 11 | Olive and canola oil | Teaspoons per day | >3 | 1-3 | <1 |
| 12 | Avocado | Units per week | >3 | 0.5-3 | <0.5 |
| 13 | Sugar | Teaspoons per day | <4 | 4-8 | >8 |
| 14 | Wine | Glasses per day | Women: ≤ 1  Men: ≤ 2  Moderate  and usually with meals | Women: ≤ 1  Men: ≤ 2  Moderate and usually with meals | Women: > 1  Men: > 2  or non-drinkers |
