# Supplementary material for: Circulating amino acids and Type 2 diabetes in a Latin American population-based cohort
Source: Cardiovasc Diabetol. 2026 Mar 26;25:116. doi: 10.1186/s12933-026-03146-8 (PMC13054996; doi:10.1186/s12933-026-03146-8)
Supplement: Supplementary file 2 — Supplementary Material 2 [file 12933_2026_3146_MOESM2_ESM.docx]

Table S2. Fully adjusted model of the association between panel of AA and T2D corrected by Bonferroni

| **Amino Acid** | **OR** | **Standard Error** | **P-value** | **CI-inferior** | **CI-superior** | **Bonferroni corrected-p** |
| --- | --- | --- | --- | --- | --- | --- |
| Alanine | 1,573339 | 0,109012 | 6,11377E-11 | 1,374534 | 1,80412 | 6,11E-10 |
| Glutamine | 0,639946 | 0,045678 | 4,00876E-10 | 0,555919 | 0,735587 | 4,01E-09 |
| Glycine | 0,682524 | 0,058405 | 8,06078E-06 | 0,574748 | 0,804015 | 8,06E-05 |
| Histidine | 0,795877 | 0,056529 | 0,001307167 | 0,692183 | 0,914561 | 0,013072 |
| Total BCAA | 2,13654 | 0,170386 | 1,73562E-21 | 1,832972 | 2,506492 | 1,74E-20 |
| Isoleucine | 1,950776 | 0,150194 | 3,98709E-18 | 1,682827 | 2,276483 | 3,99E-17 |
| Leucine | 2,173606 | 0,183321 | 3,40181E-20 | 1,848601 | 2,573602 | 3,4E-19 |
| Valine | 2,055292 | 0,156854 | 3,73503E-21 | 1,773986 | 2,393418 | 3,74E-20 |
| Phenylalanine | 1,064739 | 0,074209 | 0,36810269 | 0,926344 | 1,2183 | 1 |
| Tyrosine | 1,073837 | 0,076461 | 0,317076824 | 0,932197 | 1,232981 | 1 |

Adjusted by age, body mass index, educaton level, mediterranean diet score, smoking status, alcohol consumption and physical activity
