# Supplementary material for: Circulating amino acids and Type 2 diabetes in a Latin American population-based cohort
Source: Cardiovasc Diabetol. 2026 Mar 26;25:116. doi: 10.1186/s12933-026-03146-8 (PMC13054996; doi:10.1186/s12933-026-03146-8)
Supplement: Supplementary file 3 — Supplementary Material 3 [file 12933_2026_3146_MOESM3_ESM.docx]

Supplementary material S3 to S6

| Table S3. Association between AA at recruitment to MAUCO cohort and prevalent T2D in 1738 MAUCO participants sex strata. | | | | | |  |
| --- | --- | --- | --- | --- | --- | --- |
|  | Men OR (95% CI) | Women OR (95% CI) | Corrected P-values Men | | Corrected P-values Women | |
| **Amino Acid** |  |  | **Benjamini-Hochberg** | **Bonferroni** | **Benjamini-Hochberg** | **Bonferroni** |
| Alanine | 1.24 (1.02-1.5) | 2.01 (1.65-2.47) | 0.047 | 0.328 | 0.0 | 0.0 |
| Glutamine | 0.64 (0.52-0.78) | 0.66 (0.54-0.8) | 0.0 | 0.0 | 0.0 | 0.0 |
| Glycine | 0.7 (0.51-0.94) | 0.67 (0.55-0.82) | 0.038 | 0.225 | 0.0 | 0.001 |
| Histidine | 0.8 (0.65-0.99) | 0.79 (0.65-0.95) | 0.048 | 0.386 | 0.018 | 0.147 |
| Total BCAA | 1.83 (1.49-2.26) | 2.7 (2.13-3.49) | 0.0 | 0.0 | 0.0 | 0.0 |
| Isoleucine | 1.66 (1.38-2.02) | 2.52 (1.98-3.26) | 0.0 | 0.0 | 0.0 | 0.0 |
| Leucine | 1.83 (1.48-2.28) | 2.83 (2.19-3.71) | 0.0 | 0.0 | 0.0 | 0.0 |
| Valine | 1.81 (1.47-2.24) | 2.46 (1.97-3.09) | 0.0 | 0.0 | 0.0 | 0.0 |
| Phenylalanine | 1 (0.81-1.23) | 1.12 (0.92-1.34) | 0.975 | 1.0 | 0.26 | 1.0 |
| Tyrosine | 1.07 (0.86-1.31) | 1.09 (0.9-1.32) | 0.584 | 1.0 | 0.361 | 1.0 |

| Table S4. Association between AA at recruitment to MAUCO cohort and prevalent T2D in 1738 MAUCO participants fatty liver strata. | | | | | | |
| --- | --- | --- | --- | --- | --- | --- |
|  | Non fatty liver_1_ OR (95% CI) | Fatty liver OR (95% CI) | Bonferroni  Corrected P-values Non Fatty liver | | Bonferroni  Corrected P-values Fatty liver | |
| **Amino Acid** |  |  | **Benjamini-Hochberg** |  | **Benjamini-Hochberg** |  |
| Alanine | 1.5 (1.21-1.85) | 1.51 (1.26-1.82) | 0.0 | 0.002 | 0.0 | 0.0 |
| Glutamine | 0.72 (0.57-0.9) | 0.6 (0.49-0.72) | 0.007 | 0.044 | 0.0 | 0.0 |
| Glycine | 0.79 (0.62-1.00) | 0.65 (0.51-0.82) | 0.072 | 0.578 | 0.001 | 0.004 |
| Histidine | 0.75 (0.6-0.94) | 0.8 (0.67-0.96) | 0.02 | 0.143 | 0.019 | 0.154 |
| Total BCAA | 2.06 (1.65-2.63) | 1.99 (1.62-2.47) | 0.0 | 0.0 | 0.0 | 0.0 |
| Isoleucine | 2.14 (1.7-2.75) | 1.67 (1.38-2.04) | 0.0 | 0.0 | 0.0 | 0.0 |
| Leucine | 2.02 (1.59-2.61) | 2.07 (1.66-2.6) | 0.0 | 0.0 | 0.0 | 0.0 |
| Valine | 1.97 (1.59-2.47) | 1.95 (1.59-2.41) | 0.0 | 0.0 | 0.0 | 0.0 |
| Phenylalanine | 1.07 (0.86-1.31) | 1.03 (0.85-1.24) | 0.516 | 1.0 | 0.765 | 1.0 |
| Tyrosine | 1.11 (0.88-1.38) | 0.95 (0.78-1.15) | 0.417 | 1.0 | 0.67 | 1.0 |
| 1. Fatty liver was obtained by Ultrasound | |  |  |  |  |  |

| Table S5. Association between AA at recruitment to MAUCO cohort and prevalent T2D in 1738 MAUCO participants waist circumference obese strata. | | | | | | |  |
| --- | --- | --- | --- | --- | --- | --- | --- |
|  | Non central obesity_1_ OR (95% CI) | Central obesity_2_ OR (95% CI) | Corrected P-values Non central obesity | | Corrected P-values Central obesity | |  |
| **Amino Acid** |  |  | **Benjamini-Hochberg** | **Bonferroni** | **Benjamini-Hochberg** | **Bonferroni** |  |
| Alanine | 1.27 (0.98-1.66) | 1.74 (1.47-2.06) | 0.09 | 0.718 | 0.0 | 0.0 |  |
| Glutamine | 0.61 (0.47-0.8) | 0.66 (0.56-0.78) | 0.0 | 0.002 | 0.0 | 0.0 |  |
| Glycine | 0.72 (0.5-1) | 0.68 (0.55-0.82) | 0.086 | 0.603 | 0.0 | 0.001 |  |
| Histidine | 0.8 (0.62-1.05) | 0.79 (0.67-0.93) | 0.114 | 1.0 | 0.007 | 0.054 |  |
| Total BCAA | 2.6 (1.91-3.66) | 1.93 (1.61-2.33) | 0.0 | 0.0 | 0.0 | 0.0 |  |
| Isoleucine | 2.24 (1.7-3.05) | 1.79 (1.49-2.15) | 0.0 | 0.0 | 0.0 | 0.0 |  |
| Leucine | 2.34 (1.73-3.28) | 2.05 (1.69-2.51) | 0.0 | 0.0 | 0.0 | 0.0 |  |
| Valine | 2.62 (1.94-3.61) | 1.83 (1.55-2.19) | 0.0 | 0.0 | 0.0 | 0.0 |  |
| Phenylalanine | 1.27 (1-1.6) | 0.97 (0.81-1.14) | 0.072 | 0.434 | 0.766 | 1.0 |  |
| Tyrosine | 1.25 (0.93-1.67) | 1.00 (0.85-1.18) | 0.131 | 1.0 | 0.957 | 1.0 |  |
| 1. Waist circumference ≥102 cm in Men & ≥88 cm in Women | | |  |  |  |  |  |
| 2. Waist circumference <102 cm in Men & <88 cm in Women | | |  |  |  |  |  |
| OR was obtained from logistic regression adjusted by age, sex, education level, mediterranean diet score, smoker status, alcohol consumption and physical activity | | | | | | | |

| Table S6. Association between AA at recruitment to MAUCO cohort and prevalent T2D in 1738 MAUCO participants obese status strata. | | | | | |  |
| --- | --- | --- | --- | --- | --- | --- |
|  | BMI <30 OR (95% CI) | BMI≥30 OR (95% CI) | Corrected P-values BMI <30 | | Corrected P-values BMI≥30 | |
| **Amino Acid** |  |  | **Benjamini-Hochberg** | **Bonferroni** | **Benjamini-Hochberg** | **Bonferroni** |
| Alanine | 1.73 (1.43-2.1) | 1.45 (1.2-1.77) | 0.0 | 0.0 | 0.0 | 0.002 |
| Glutamine | 0.69 (0.57-0.84) | 0.6 (0.49-0.73) | 0.0 | 0.003 | 0.0 | 0.0 |
| Glycine | 0.72 (0.56-0.89) | 0.63 (0.49-0.81) | 0.006 | 0.039 | 0.0 | 0.003 |
| Histidine | 0.81 (0.66-0.99) | 0.77 (0.63-0.93) | 0.038 | 0.381 | 0.01 | 0.083 |
| Total BCAA | 2.75 (2.19-3.53) | 1.67 (1.36-2.08) | 0.0 | 0.0 | 0.0 | 0.0 |
| Isoleucine | 2.52 (2.02-3.19) | 1.5 (1.23-1.86) | 0.0 | 0.0 | 0.0 | 0.001 |
| Leucine | 2.91 (2.27-3.79) | 1.66 (1.33-2.08) | 0.0 | 0.0 | 0.0 | 0.0 |
| Valine | 2.49 (2.01-3.12) | 1.69 (1.38-2.09) | 0.0 | 0.0 | 0.0 | 0.0 |
| Phenylalanine | 1.23 (1.02-1.48) | 0.93 (0.76-1.13) | 0.031 | 0.251 | 0.551 | 1.0 |
| Tyrosine | 1.25 (1.02-1.52) | 0.99 (0.82-1.19) | 0.034 | 0.302 | 0.928 | 1.0 |
